# Deconvolution of the tumor-educated platelet transcriptome reveals activated platelet and inflammatory cell transcript signatures

**DOI:** 10.1172/jci.insight.178719

**Published:** 2024-08-27

**Authors:** Jerome M. Karp, Aram S. Modrek, Ravesanker Ezhilarasan, Ze-Yan Zhang, Yingwen Ding, Melanie Graciani, Ali Sahimi, Michele Silvestro, Ting Chen, Shuai Li, Kwok-Kin Wong, Bhama Ramkhelawon, Krishna P.L. Bhat, Erik P. Sulman

**Affiliations:** 1 Department of Radiation Oncology, NYU Grossman School of Medicine, New York, New York, USA.; 2 Department of Radiation Oncology, Keck School of Medicine of University of Southern California, Los Angeles, California, USA.; 3 Department of Cell Biology and; 4 Laura and Isaac Perlmutter Cancer Center, NYU Grossman School of Medicine, New York, New York, USA.; 5 Department of Cancer Biology, Mayo Clinic, Scottsdale, Arizona, USA.

**Keywords:** Oncology, Brain cancer, Platelets

## Abstract

Tumor-educated platelets (TEPs) are a potential method of liquid biopsy for the diagnosis and monitoring of cancer. However, the mechanism underlying tumor education of platelets is not known, and transcripts associated with TEPs are often not tumor-associated transcripts. We demonstrated that direct tumor transfer of transcripts to circulating platelets is an unlikely source of the TEP signal. We used CDSeq, a latent Dirichlet allocation algorithm, to deconvolute the TEP signal in blood samples from patients with glioblastoma. We demonstrated that a substantial proportion of transcripts in the platelet transcriptome are derived from nonplatelet cells, and the use of this algorithm allows the removal of contaminant transcripts. Furthermore, we used the results of this algorithm to demonstrate that TEPs represent a subset of more activated platelets, which also contain transcripts normally associated with nonplatelet inflammatory cells, suggesting that these inflammatory cells, possibly in the tumor microenvironment, transfer transcripts to platelets that are then found in circulation. Our analysis suggests a useful and efficient method of processing TEP transcriptomic data to enable the isolation of a unique TEP signal associated with specific tumors.

## Abstract 2

A machine learning algorithm suggests that platelets in patients with cancer appear to ingest RNA from the inflammatory cells in the tumor’s environment.

## Graphical abstract

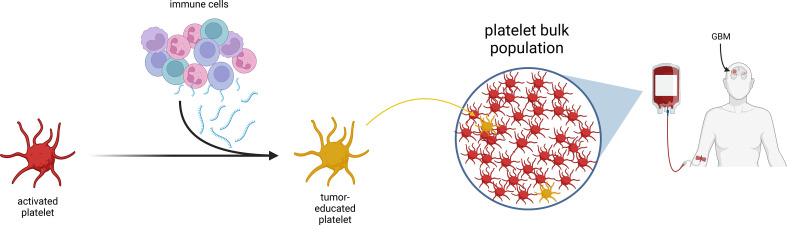

## Introduction

Liquid biopsy has garnered increasing interest as a method of interrogating tumors before treatment, immediately after treatment to test for minimal residual disease, after treatment to assess for recurrence, and even before diagnosis as a screening method (1–3). Several liquid biopsy methods have been proposed and developed, including circulating tumor cells, circulating tumor DNA, and extracellular vesicles. These techniques may identify the presence of tumors, may characterize their biological properties, and may be used to tailor therapy precisely to the molecular features of the cancer cells. Furthermore, liquid biopsy may offer advantages over direct tumor biopsy, beyond the obvious technical advantage that liquid biopsy is substantially less invasive. For example, direct biopsy of the tumor does not always reflect tumor heterogeneity if the biopsy procedure samples only part of the tumor, whereas liquid biopsy is more likely to account for tumor heterogeneity, potentially allowing for further tailoring of therapy (4).

For primary brain tumors, however, traditional liquid biopsy methods are not sufficiently effective (5). One report noted that fewer than 10% of patients with glioma had detectable circulating tumor DNA (6). Similarly, exosomes can aid in the detection of epidermal growth factor receptor variant III, a molecular alteration found in about 30% of glioblastomas, but with specificity and sensitivity comparable to those of brain MRI (7). The inability to detect primary brain tumors using liquid biopsy presents an especially acute problem, because many primary brain tumors have high rates of recurrence (8). Glioblastoma (GBM), the most common and lethal primary brain tumor in adults, has a median survival of 21 months even with standard-of-care treatment, including resection, adjuvant radiotherapy with concurrent and adjuvant temozolomide, and tumor-treating fields (9). Tumor recurrence is generally considered inevitable but is difficult to detect correctly because of the phenomenon of “pseudoprogression,” in which subacute effects of radiotherapy and chemotherapy can imitate the appearance of an enlarging, recurrent tumor on MRI (10, 11). This highlights the need for accurate methods to detect the presence or recurrence of high-grade brain tumors through methods that may complement imaging, such as liquid biopsy.

A recent report by Sol et al. demonstrated the feasibility of using “tumor-educated platelets” (TEPs) as a liquid biopsy for GBM (12). Multiple studies have demonstrated that the platelet transcriptome in patients with cancers of multiple origins differs from that of healthy controls (13–15). Several mechanisms have been proposed for “tumor education,” including the suggestion that the tumor transfers mRNA transcripts directly to tumor cells (14, 16), that patients with cancer have preferential upregulation of reticulated platelets with differing transcriptomes (17), or that the tumor produces factors that alter platelet mRNA splicing and thus modulate gene expression (18). However, none of these mechanisms have been definitively demonstrated, limiting the ability to precisely define the TEP signal and develop its use as an assay with clinical utility in detecting initial tumor burden, minimal residual disease, or tumor recurrence. Studies of TEPs in the clinical sphere have focused on using supervised machine learning algorithms to provide a clinically utilizable computational model (12, 17). For example, Sol et al. identified 203 genes that constitute a TEP signal that distinguishes platelets in patients with GBM from platelets in healthy controls. However, these genes were not established as glioma related. Furthermore, many high-ranking genes in the signal, such as *CA1*, *CD163*, and *S100A12*, are typically associated with nonplatelet circulating cells, further raising the question of what mechanism drives the expression of these transcripts in platelets. In this study, we used an interpretable, unsupervised machine learning algorithm to elucidate the potential mechanism underlying tumor education of platelets.

## Results

**Figure 1:**
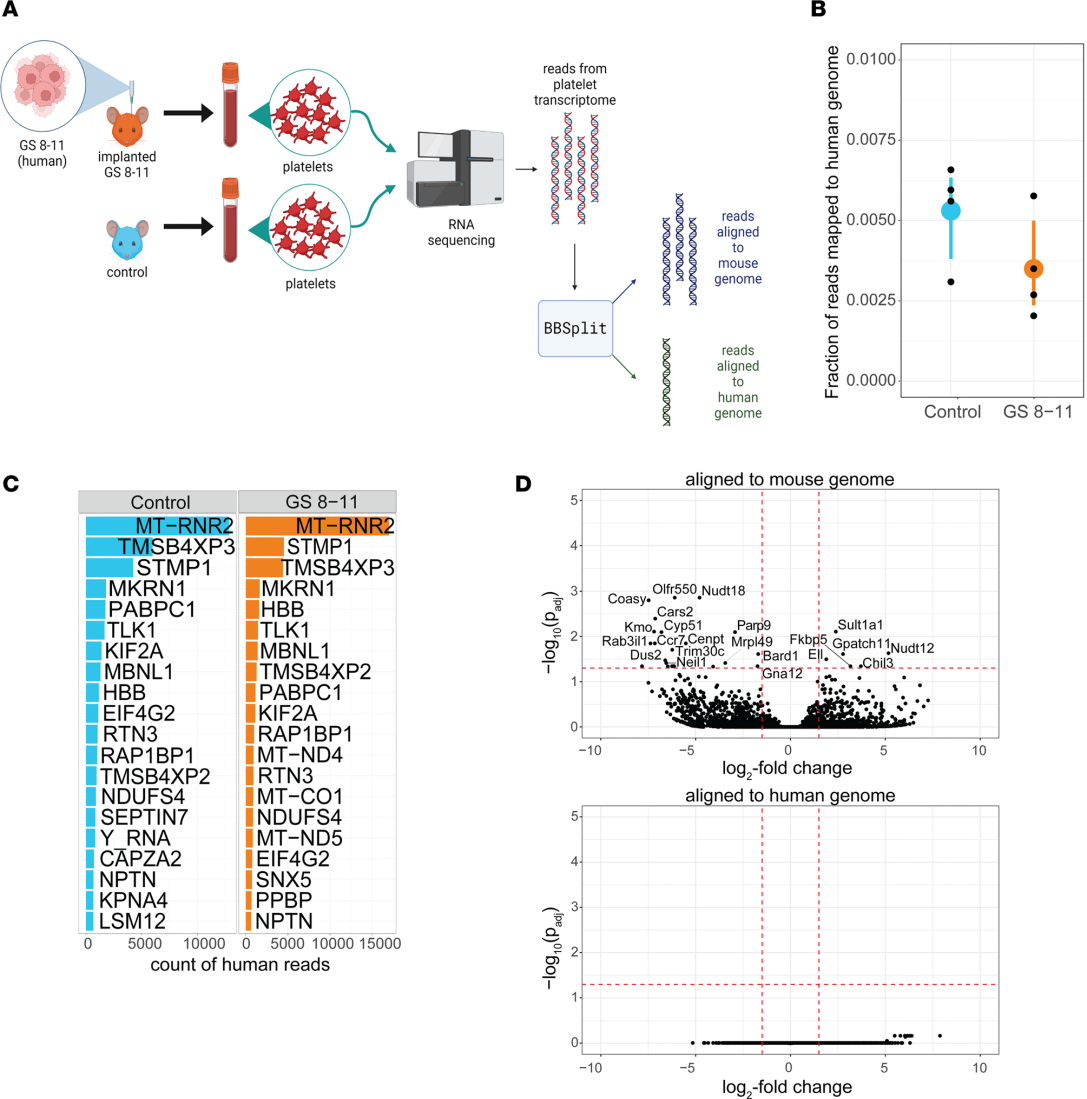
Analysis of platelets from mouse implanted with a human brain tumor. (**A**) Schematic of experiment. Mice are implanted with cells from human GS 8-11 line, and then mouse platelets are harvested from implanted mice and control mice, for downstream RNA-Seq. Created by BioRender.com. (**B**) Fraction of reads in each sample mapped to the human genome hg38 for control mice and mice implanted with GS 8-11 tumors (each *n* = 4). The large colored dot represents the mean value, with lines extending from the mean representing 95% confidence intervals calculated using a bootstrap. (**C**) The top 20 genes mapped to the human genome for platelets from control mice and platelets from mice implanted with GS 8-11 tumor. (**D**) Differential expression of mouse-aligned and human-aligned genes using DESeq2, with significantly altered expression (*P* < 0.05, Wald’s test) highlighted.

To test the hypothesis that platelets receive RNA transcripts directly from the tumor, we implanted tumors derived from the GS 8-11 glioma stem cell line into nude mice and drew platelet samples from these mice. The platelet transcriptomes of these mice were compared with those of control mice using the BBSplit tool (19) to isolate transcripts mapping to the human genome (hg38) from those mapped to the mouse genome (mm10) (Figure 1A). An average of 0.35% of transcripts from the platelets of the tumor-implanted mice were mapped to the human genome, compared with 0.53% of transcripts from the platelets of the control mice (Figure 1B). Based on these results, it appears unlikely that there is direct transfer of transcripts from the tumor to circulating platelets. Furthermore, the identities of transcripts mapped to the human genome were predominantly similar between the tumor-implanted and control mice (Figure 1C), suggesting that these are not truly human transcripts but reflect incorrect mapping of these mouse transcripts to the human genome. Additionally, differential expression testing using DESeq2 (20) showed that no transcript mapping to the human genome was significantly upregulated in the tumor-implanted mice, though several transcripts mapped to the mouse genome were significantly upregulated or downregulated in the tumor-implanted mice (Figure 1D).

**Figure 2:**
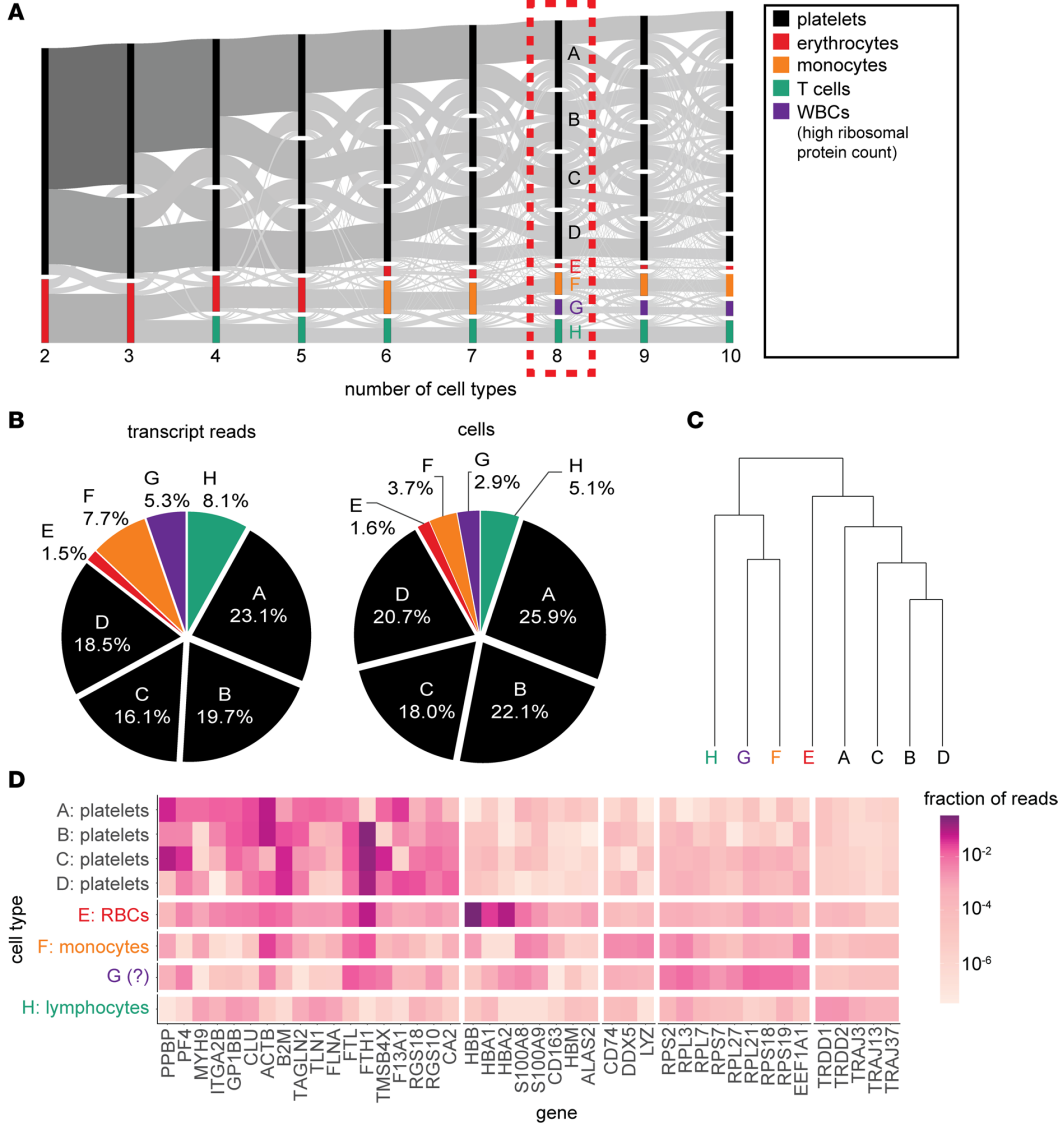
Deconvolution of the platelet transcriptome using CDSeq. (**A**) Results of the deconvolution algorithm, using varying numbers of cell types (ranging from 2 to 10 cell types). Each column represents the same set of reads deconvoluted into a different number of cell types. Flow from one column to the next represents an estimate of the repartitioning of reads into a larger number of cell types. For the remaining figures, we use the deconvolution into 8 cell types. (**B**) The proportion of each cell type with regard to total number of transcript reads (left) and total number of cells (right). (**C**) Clustering of cell types based on similarity. Distance is computed by taking Spearman’s correlation coefficient between 2 cell type gene expression profiles and converting it from a value ranging from 1 to –1, to a distance in [0,1]. (**D**) Prominent genes found in each cell type with darker color corresponding to higher fraction of reads of the given gene in the given cell type.

Given that the direct transfer of RNA from the tumor to circulating platelets appears unlikely, we attempted to find alternative explanations for the observed changes in the platelet transcriptome associated with GBM. Since many of the transcripts included in the TEP signature are not typically associated with the platelet transcriptome, we hypothesized that the platelet transcriptome–sequencing data require further processing to remove contaminant transcripts that are not platelet derived and to isolate the correct signature associated with the presence of tumor. We used the program CDSeq (21) to deconvolute the platelet transcriptome in an unbiased manner using an unsupervised machine learning algorithm based on latent Dirichlet allocation (LDA; see Methods). The algorithm takes the read counts from a group of RNA-Seq experiments as input and deconvolutes them into read counts for individual cell types. We used published data of platelet transcriptomes of healthy controls and patients with GBM (12), with the entire dataset, containing both patients with GBM and healthy controls, used as input to the algorithm. Because the number of cell types must be provided as input but was not known a priori, we ran the algorithm for varying numbers of cell types from 2 to 60 (Supplemental Figures 1 and 2; supplemental material available online with this article; https://doi.org/10.1172/jci.insight.178719DS1). Although the log-posterior of the output continued to increase with additional cell types, the number of cell types corresponding to nonplatelets did not increase substantially after 10 cell types. The results of the deconvolution algorithm using 2–10 cell types are presented in Figure 2A. Annotation of cell types was performed by correlating the gene expression profile of each cell type with published annotated single-cell RNA-Seq gene expression profiles (22) (see Supporting Data Values). Multiple related single cell types (e.g., CD4^+^ T cells, CD8^+^ T cells, and other T cells) were frequently highly correlated with a bulk cell type, in which case the most general cell type annotation is shown. We note that when dividing the read counts into 2 cell types, the smaller fraction appears to correspond to nonplatelets, and this nonplatelet cell type continues to be subdivided further by increasing the number of cell types. Importantly, 21.9% of the total reads in these platelet samples were derived from nonplatelet cell types, based on the division of the transcriptome into platelet and nonplatelet fractions.

We arbitrarily chose to examine the results using 8 cell types because with more cell types, there was little change in the nonplatelet cell types, and further divisions only served to increase the number of platelet cell types (Supplemental Figure 1A). The relative abundances of these cell types in all the samples are shown in Figure 2B. Notably, because the amount of RNA in platelets is substantially lower than that in other cell types in peripheral blood, the percentage of *cells* that were nonplatelets in these samples was lower than the percentage of *reads* that were not platelet derived. Clustering of the cell types based on gene expression profiles (Figure 2C) also demonstrated that cell types annotated as platelets (A, B, C, and D) were more similar to one another than the other cell types and together formed a distinct clustering branch from the nonplatelet cell types. Figure 2D shows a representation of the gene expression in each of the 8 cell types. The nonplatelet cell types are seen clearly to be nonplatelets, with the cell type annotated as “erythrocytes” (cell type E) having high levels of hemoglobin reads (*HBB*, *HBA1*, *HBA2*), the cell type annotated as “T cells” (cell type H) having an overrepresentation of T cell receptor reads (T cell receptor diversity domains and T cell receptor-α joining domains), and the cell type annotated as monocytes enriched in *LYZ*, *CD74*, and *DDX5*. Cell type G likely represents a subset of leukocytes because, upon examination of the diagram in Figure 2A, the transcripts in cell type G trace back to a common larger cell type that includes other white blood cells; additionally, this cell type contains a high proportion of ribosomal protein RNAs (e.g., *RPL7* and *RPS18*), which are considerably more abundant in nonplatelet circulating cells compared with platelets, as seen in single-cell data sets (Supplemental Figure 3). We also found that the proportion of nonplatelet transcripts was considerably higher in samples derived from specific institutions (Supplemental Figure 4), suggesting that the presence of nonplatelet transcripts in the sample might be technique dependent. For example, insufficient isolation of platelets by centrifugation, lysis of nonplatelet cells, or release of their RNA during preparation may lead to contamination of platelet RNA. This may also partially account for the batch effects reported in tumor-educated platelet collections between hospitals (23). Of note, the protocol used for the platelet samples analyzed in this study did not apply leukodepletion during platelet purification (23), whereas other protocols do include this step (24, 25), which may indicate that platelet samples in which leukodepletion is performed may have lower rates of these cell types.

**Figure 3:**
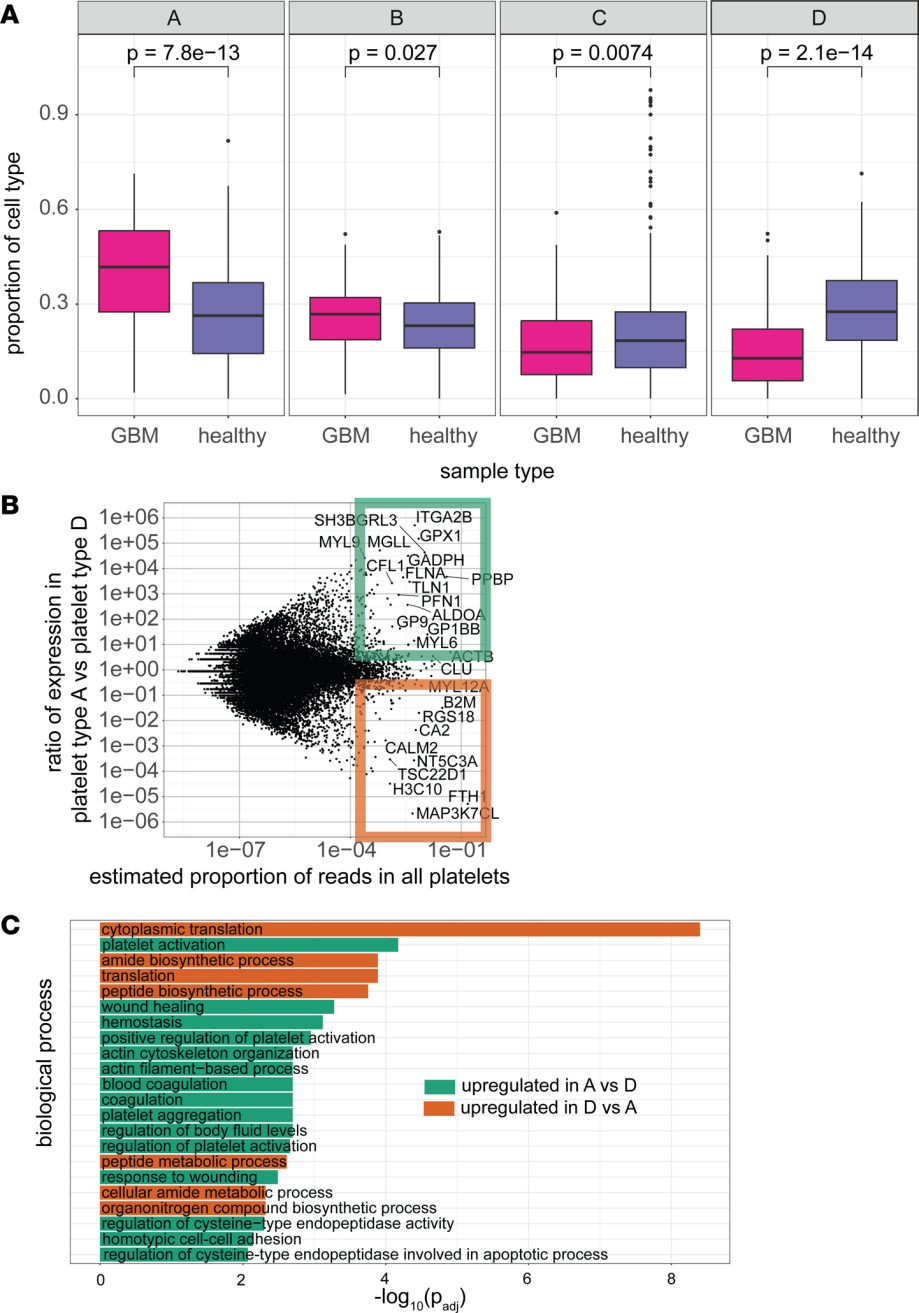
Features of platelets found in controls and patients with GBM. (**A**) Box plots showing fraction of cell types A, B, C, and D in samples from healthy controls and samples from patients with GBM, after other cell types have been removed. Means were compared via a Wilcoxon test. Box plots show the interquartile range, median (line), and minimum and maximum (whiskers). (**B**) Plots of genes in cell types A and D, with *x* axis representing the estimated representation of the gene in the 2 cell types combined and *y* axis representing the ratio of expression in cell type A to cell type D. Genes in the green and orange squares are taken to be upregulated and downregulated, respectively. (**C**) Gene Ontology terms overrepresented or underrepresented in genes that are upregulated in cell type A compared with genes that are downregulated in cell type A.

We then examined how the 4 platelet types were represented in the platelets of patients with GBM compared with platelets in healthy controls. A representation of the different platelet types in healthy controls and patients with GBM is shown in Figure 3A. We note that the largest differences were seen in platelet types A and D, where patients with GBM had significantly higher levels of platelet type A and significantly lower levels of platelet type D. We selected genes that constituted more than 0.01% of reads and were upregulated or downregulated at least 10-fold in cell type A compared with cell type D (Figure 3B). We then identified Gene Ontology (GO) biological processes that were differentially enriched in upregulated genes compared with downregulated genes (26–28) as shown in Figure 3C. Given that many of the GO terms are associated with platelet activation (platelet activation, positive regulation of platelet activation, wound healing, hemostasis) or cytoskeletal element function, which is important in platelet shape regulation after activation, we infer that cell type A represents activated platelets, while cell type D represents quiescent platelets. For the same reason, it is unsurprising that cell type D contained increased levels of *RGS10* and *RGS18*, both of which encode proteins associated with inhibition of platelet activation. (29) Cell types B and C had intermediate levels of platelet activation–related genes and *RGS10* and *RGS18* (Supplemental Figure 5), suggesting that they represent platelets with intermediate levels of activation between platelet types A and D. We refer to the transcriptomes of these subtypes as Plt_activated_ and Plt_quiescent_, respectively. Given the concern that higher levels of activated platelets might reflect increased platelet production and higher levels of reticulated platelets, we divided the dataset into samples with high levels of circular RNAs (circRNAs; greater than the median value) and low levels of circular RNAs (less than the median value), using predicted circRNA levels from the PTESFinder algorithm (30). Since circRNA levels are increased in older platelet samples, because of preferential decay of linear mRNAs over circRNAs (31), this should be a surrogate marker for overall platelet age. There was no significant difference between the fraction of circRNAs in samples from controls versus samples from patients with GBM (*P* = 0.07). Furthermore, our results were similar for the entire dataset as a whole compared with either half of the dataset (Supplemental Figure 6), suggesting that the higher levels of activated platelets are not strictly a function of increased or decreased platelet production.

**Figure 4:**
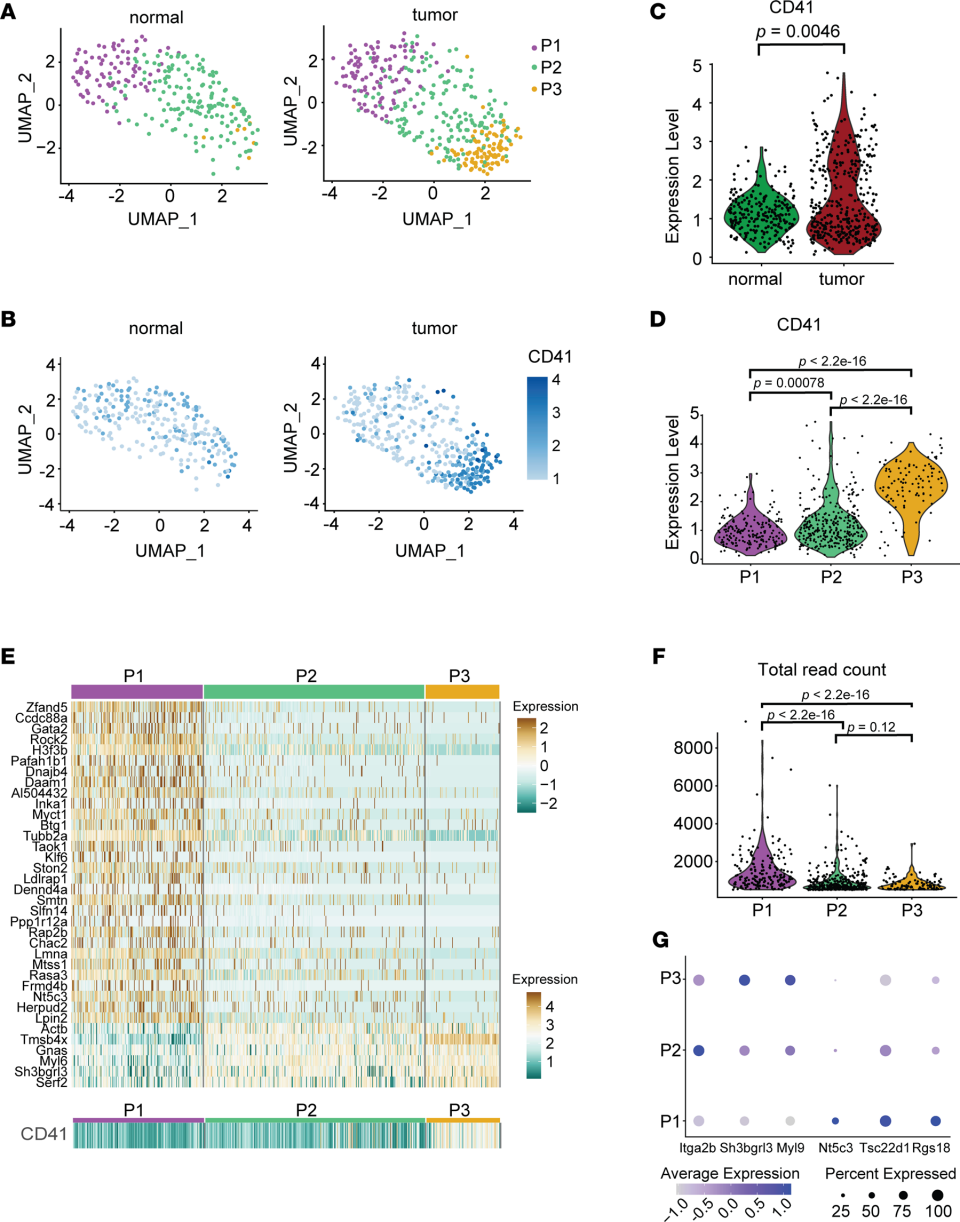
Analysis of CITE-Seq data from mouse platelets. (**A**) Uniform manifold approximation and projection (UMAP) plot of platelets collected from normal mouse and mouse implanted with tumor, with clusters P1, P2, and P3 highlighted. (**B**) CD41 surface expression in platelets. (**C** and **D**) Expression of CD41 in platelets from normal and tumor-implanted mice, and in clusters P1, P2, and P3, with *P* values from Wilcoxon’s test shown. (**E**) Gene expression heatmap for P1, P2, and P3 clusters, with genes highly expressed in P2/P3 versus P1 or vice versa shown. CD41 expression heatmap in P1, P2, and P3 clusters shown below gene expression heatmap. (**F**) Total read count per cell in clusters P1, P2, P3; *P* value using Wilcoxon’s test. Data points are shown in a jitter plot (set *y* value with random *x* value) with a superimposed violin plot showing the density along the *y* axis. (**G**) Dot plot showing expression of platelet activation–related genes (*Itga2b*, *Sh3bgrl3*, and *Myl9*) and genes associated with decreased platelet activation (*Nt5c3*, *Tsc22d1*, and *Rgs18*).

To demonstrate that changes in the transcriptional profile of these subpopulations correlate with differences in the platelet phenotype, we performed single-cell RNA-Seq with mouse whole blood using CITE-Seq (32), using peripheral blood from 1 healthy control mouse and 1 mouse with an implanted non–small cell lung cancer (NSCLC) tumor. After identifying the subset of data corresponding to platelets and clustering the platelets based on gene expression, 3 clusters of platelets were identified, which we name P1, P2, and P3 (Figure 4A). P3 appeared to be a subcluster of P2 and was predominantly observed in platelets from the tumor-bearing mouse. Platelets in cluster P3 had especially high levels of CD41 expression (Figure 4, B–D), suggesting increased platelet activation (33–35). When examining the P1, P2, and P3 clusters at the transcriptomic level (Figure 4E), P2 and P3 together had an altered transcriptional profile compared with P1, with genes highly expressed in P2 and P3, including those seen at higher levels in Plt_activated_ than in Plt_quiescent_, such as *Myl6*, *Sh3bgrl3*, and *Actb*. Cluster P1 contained higher levels of genes associated with megakaryocytes, including *Rock2* (36), *Daam1* (37), and *Gata2* (38), as well as genes previously associated with “young” platelets such as *H3f3b* (39), suggesting that these represent young platelets that have more recently been released from megakaryocytes and are thus less likely to have been activated. P1 also contained higher total read counts (Figure 4F), which is consistent with this cluster representing younger platelets, since older platelets have undergone mRNA decay and cannot synthesize new mRNA since they are anucleate. P2 and P3 had higher levels of transcripts associated with platelet activation, including *Itga2b*, *Sh3bgrl3*, and *Myl9*; lower levels of *Rgs18*, which is associated with negative regulation of platelet activation; and lower levels of *Nt5c3* and *Tsc22d1*, which were highly expressed in the Plt_quiescent_ subtype (Figure 4G).

**Figure 5:**
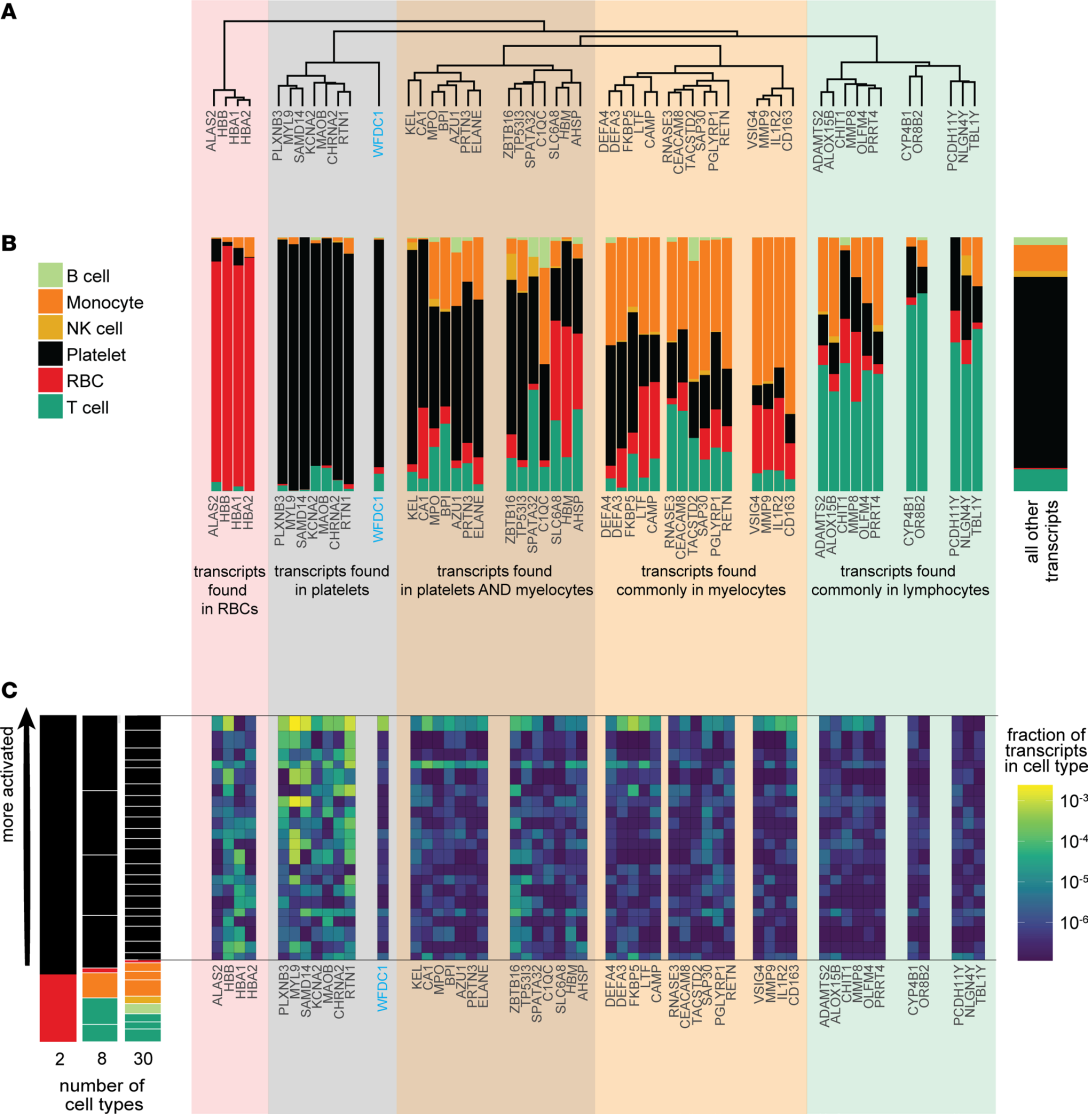
Genes upregulated in GBM-associated platelets. (**A**) Clustering dendrogram for genes upregulated in platelets of patients with GBM. (**B**) Distribution of these genes among cell types when deconvoluting into 30 cell types. Distribution of all other genes among cell types is shown on the right side. (**C**) Expression of these genes among platelet cell types. Platelet cell types are ordered vertically by finding the Hellinger distance of the platelet type gene expression from the Plt_activated_ transcriptome, such that platelet types at the top are most similar to Plt_activated._

We then returned to analyze the platelet samples collected from control patients and patients with GBM, analyzing the cell type distribution of genes that were differentially upregulated in the platelet transcriptome of patients with GBM. We selected genes with greater than 1.25 log_2_-fold upregulation. Importantly, these genes did not include transcripts known to be upregulated in GBM cells. These were then clustered using a hierarchical clustering algorithm (see Methods); the clustering results are shown in Figure 5A. We then identified the distribution of these transcripts by deconvolution into 30 cell types (Figure 5B). Some of these transcripts are heavily represented in platelet cell types, whereas others are mostly represented in nonplatelet cell types, including red blood cells, monocytes, and T cells. Figure 5C shows the expression of these transcripts in each platelet cell type. GBM-associated transcripts that were mostly expressed in platelets with little expression in nonplatelets were expressed in many platelet cell types, with an apparent preference for activated platelet cell types (those that are most similar to Plt_activated_). This suggests that these genes were upregulated in the platelet transcriptome of patients with GBM, as these patients had increased levels of activated platelets, as noted previously.

When examining GBM-associated transcripts that were mostly *not* expressed in platelets, as shown in Figure 5B, those that were mostly expressed in monocytes or T cells did not appear in most platelet cell types, including most activated platelet cell types. Rather, they were preferentially expressed in one of the platelet cell types, corresponding to the most highly activated platelet type. This suggests that these genes are mostly expressed in *non*platelets but are also found in a small subset of activated platelets, which may indicate that these transcripts originate in circulating nonplatelet cells and are then transferred to a small fraction of platelets. Of note, genes that are upregulated approximately equally in platelets, monocytes, and T cells had especially high expression in this one platelet subtype, and then lower expression in other activated platelets, suggesting that these are genes expressed in activated platelets, but also in nonplatelet cells, which may then transfer some of these transcripts to platelets. GBM-associated transcripts that are expressed in red blood cells appear to be expressed in all activated platelet cell types, similar to transcripts that are predominantly expressed in platelets. This likely represents the fact that red blood cells adhere directly to platelets to promote aggregation and degranulation (40), such that activated platelet cell types contain transcripts originating from red blood cells that are bound to platelets. Only 1 gene, *WFDC1*, was expressed predominantly in platelets and not in other cell types, but it also appeared only in 1 activated platelet type where these other genes are expressed. Of note, this activated platelet type was specific to GBM-associated platelets and not other inflammatory conditions; analysis of the different platelet subtypes in samples from patients with a nonneoplastic inflammatory condition, multiple sclerosis, did not show elevation of the GBM-associated platelet subtype or elevation of the Plt_activated_ cell type more generally (Supplemental Figure 7).

This analysis suggests that the deconvolution method used here could be employed in a *supervised* manner to predict whether tumor-educated platelets from patients with GBM are present. Indeed, we were able to reanalyze these data using a supervised LDA algorithm (41), splitting the dataset into training (80%) and validation (20%) subsets and using the algorithm to predict whether a sample represents a control patient or a patient with GBM using cell type composition. The algorithm successfully differentiated GBM samples from control samples (training: AUC 0.84, validation: AUC 0.83) (Supplemental Figure 8), using only cell type composition as a predictor variable and without direct use of read counts as predictors (see Methods). This suggests that cell type composition, including presence of activated platelets and platelets containing ingested mRNAs, could be directly used for sample prediction.

To address the concern that TEPs might not contain platelet-related reads because of the primary malignancy being protected by the blood-brain barrier, we repeated the analysis for a second malignancy, NSCLC. Indeed, in NSCLC, we found a similar pattern of findings. We again found that a substantial fraction of reads in analyzed blood samples did not correspond to platelets but to other cell types, including erythrocytes, leukocytes, and monocytes (Supplemental Figure 9). When analyzing the cell types generated when deconvoluting the samples into 7 distinct cell types, 4 of these types corresponded to platelets. Two of these cell types were significantly enriched in samples from patients with NSCLC, while a third cell type was significantly decreased in these patients (Supplemental Figure 10). The NSCLC-enriched fractions had similar profiles to the GBM-enriched fraction of platelets, corresponding to the activated subtype, with high levels of *ACTB*, *ITGA2B*, *MYL6*, and *GP1BB*; the NSCLC-depleted fraction had high levels of *RGS18* similar to the GBM-depleted fraction. Similar to GBM, the genes highly expressed in platelets from patients with NSCLC included those predominantly expressed in platelets, which were mostly seen in activated platelet subtypes, and those predominantly expressed in nonplatelets, which were confined to 1–2 platelet subtypes (Supplemental Figure 11). Our analysis of the NSCLC platelet samples also allowed for comparison with samples drawn from patients with metastatic NSCLC to the brain (Supplemental Figure 12). Of note, these samples had elevated levels of 1 of the 4 platelet subtypes, similar to primary NSCLC samples, corresponding to activated platelets. Additionally, when these samples from patients with brain metastases were deconvoluted into 30 subtypes, a subtype that was highly enriched in samples from patients with primary NSCLC, with high levels of *IFITM3*, *IFI27*, *TPM2*, and *HBG2* reads, was similarly enriched in samples from patients with brain metastases.

## Discussion

Tumor education of platelets has eluded mechanistic interpretation, hampering further development and use of this liquid biopsy method. Our analysis suggests that circulating platelets likely do not directly receive mRNA transcripts from the tumor. Instead, the TEP signal is found in a minority of platelets whose mRNA can be separated using computational means from a much larger sample of platelet mRNA. Our results suggest that a non-negligible fraction, approximately 20%, of the “platelet” transcriptome is in fact derived from nonplatelet cells, serving to contaminate the transcriptome. If these transcripts are removed, the remaining transcripts fall on a spectrum from quiescent to activated platelets, and the presence of GBM increases the rate of activated circulating platelets.

Activated platelets have increased levels of transcripts, including *ITGA2B*, *MYH9*, and *ACTB*, which are associated with the process of platelet activation, and lower levels of transcripts, including *RGS18*, which are associated with decreased platelet activation. Furthermore, a small subset of activated platelets contains mRNAs that constitute biomarkers that represent the presence of tumor. The latter mRNAs appear to be those that are also found primarily in nonplatelet cells, including erythrocytes, leukocytes, and myelocytes. Such nonplatelet cells may interact with activated platelets in the tumor microenvironment, transferring inflammatory markers that can then be directly detected in the transcriptome of circulating platelets (42). This explanation is consistent with the finding that none of the TEP markers are transcripts known to be upregulated within the tumor itself. Furthermore, previous reports have suggested that the TEP signal reflects activated platelets (17), but transcripts representative of platelet activation, such as *ITGA2B*, have not been identified by supervised machine learning algorithms as part of the signature used to classify platelets as tumor educated or control. Our analysis suggests that platelet activation is permissive of the TEP signal, but not sufficient, and that the TEP signal is contained in only a small fraction of activated platelets. We do not see a clear difference in the signature between localized disease and metastatic disease (in the case of NSCLC); this may reflect that our analytic method is not sensitive enough to distinguish such a difference or that the TEP signal is only produced in a small fraction of platelets such that extent of disease could not be expected to increase the intensity of the signal.

Our analysis also suggests that the removal of nonplatelet transcripts is important for the detection and interpretation of the TEP signal. Other reports have analyzed the presence of ribosomal protein transcripts or hemoglobin transcripts found in the platelet transcriptome associated with tumor or benign conditions (43–45). However, we suggest that most of these transcripts originate from nonplatelet cells in the blood that are lysed during sample processing. On the other hand, the small subset of activated platelets with TEP signal-associated transcripts contains transcripts that were seen mostly in monocytes and lymphocytes. That is, in addition to transcripts in the sample that indeed derived from nonplatelets, platelet samples from patients with GBM also contain platelet-derived transcripts that are likely to have *originated* in nonplatelets. One possibility to explain this finding is that these transcripts may be transferred to platelets from monocytes in the tumor microenvironment, either actively or passively, and may represent a useful biomarker of tumor presence. *WFDC1* is the only gene that is upregulated in GBM-associated platelets and is expressed predominantly in platelets but appears only in a small subset of platelets associated with the TEP signal. This suggests that *WFDC1* transcripts may originate from other nonblood cells, perhaps in the tumor microenvironment, and are then transferred to this subset of platelets. Notably, endothelial expression of *WFDC1* is found in the brain vasculature comprising the blood-brain barrier in mice, where it has been found to regulate inflammation and wound repair (46). At sites of blood-brain barrier disruption, such as gliomas, *WFDC1* may be highly expressed among endothelial cells interacting with platelets.

Deconvolution of platelet transcriptomic data may serve as a sort of computational “filter” to isolate platelet mRNA, which can then be analyzed further for signals representative of tumor presence. One notable limitation of this technique is the substantial computational time required to employ this deconvolution algorithm, which, as a Gibbs Monte Carlo Markov chain algorithm, is not parallelizable. Other deconvolution algorithms that use simpler techniques, such as non-negative matrix factorization, are expected to be quicker but may also be less accurate in removing nonplatelet transcripts. Furthermore, a notable advantage of the CDSeq algorithm is that no reference cell types are required, which is important because the composition of different platelet cell types is not well defined.

We were unable to ascertain the definitive origin of the TEP signal transcripts, though, as noted, they are not likely to be derived directly from tumor cells, as they are not considered representative transcripts of the tumor and are more likely to be derived from other nontumor cells, perhaps from supporting cells in the tumor microenvironment. Bidirectional biomolecular transfer is well established between platelets and other cells, including endothelial cells and inflammatory cells (47). Thus, the increased activation of platelets in patients with GBM may benefit the tumor by allowing the transfer of RNAs to and from platelets. This may then be exploited for clinical benefit by detecting activated platelets in which mRNA has been transferred from cells in the tumor microenvironment. Because the tumor microenvironment differs between tumors, the biomolecules abundant in the microenvironment, as well as those most likely to be detected in circulating platelets, are expected to differ as well, as evidenced by reports showing different TEP signals for different cancers. Further studies using deconvoluted transcriptomic data from platelets in patients with various tumors may further elucidate the inflammatory markers unique to each tumor.

Overall, we find that the use of an unsupervised machine learning algorithm for deconvolution allows for further mechanistic insight into the nature of the tumor-educated platelet signal. Although complete insight into the origin of tumor-educated platelet transcripts cannot be provided by computational insights alone, further experimental work may be able to trace transcripts, possibly originating from inflammatory cells, transferred to circulating platelets. The signature produced will benefit from additional validation in a prospective study of a patient population with GBM. This would allow for analysis of other patient markers that may correlate with inflammatory transcripts seen in the TEP data, such as erythrocyte sedimentation rate, and other clinical variables, such as smoking and medications, that might alter platelet states. The analytic pipeline used in this study may allow for decontaminated transcriptomic data, which may better elucidate the role of these variables in platelet composition.

## Methods

### Sex as a biological variable.

Sex was not considered as a biological variable in analysis of human samples, which included both men and women. Mouse experiments were performed exclusively on female mice, and it is unknown whether the findings are relevant for male mice.

### Deconvolution with CDSeq.

FASTQ files containing RNA-Seq data were obtained from the National Center for Biotechnology (NCBI) Gene Expression Omnibus (GEO) database under accession number GSE156902 (12). Raw FASTQ files were processed using Trimmomatic version 0.36 (48) for trimming and clipping sequence adapters. The reads were then aligned to the human reference hg38 genome using STAR (49), and the aligned reads were summarized using HTSeq 0.13.5 (50). The dataset for analysis of the GBM TEP profile included all normal samples and all GBM samples, excluding follow-up specimens. Levels of circRNA in each sample (discussed in Supplemental Figure 6) were predicted by using PTESFinder (30). Aligned reads from the same specimen stored in separate files were combined. Genes with fewer than 400 reads across all the samples were removed from the analysis.

The resulting dataset contained 437 samples, including 89 samples from patients with GBM, 348 healthy controls, and 20,367 genes. Mitochondrial reads were removed from the dataset. This dataset was then provided as input to CDSeq (21), using the parameters α = 5 and β = 0.5. Results of deconvolution with varying values of α and β were substantively similar (Supplemental Figure 13). To make the process computationally tractable to run with different numbers of cell types, we used the CDSeq data dilution module with a dilution factor of 10. We then ran CDSeq on the dataset for each number of cell types in the range of 2 to 60 to determine the ideal number of cell types. Each run was performed for 1,000 Markov chain Monte Carlo steps.

To analyze the relationship between cell types in each run, we tracked the cell types to which the reads of a given gene/sample combination were assigned. For example, suppose the number of reads of gene *g* in sample *s* that are assigned to cell type *t* among *T* total cell types (*t* = 1, 2, 3, …, *T*) is given by 
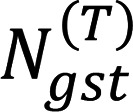
, so that the total number of reads of gene *g* in sample *s* is 
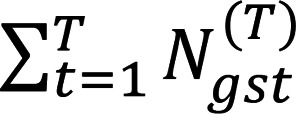
. Also denote the number of reads of gene *g* in sample *s* that are assigned to cell type *t*′ among *T* + 1 cell types (*t* = 1, 2, 3, …, *T*, *T* + 1) by 
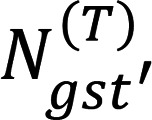
.

Now, we can estimate the number of reads of gene *g* in sample *s* assigned to cell type *t* among *T* cell types that are also assigned to cell type *t*′ among *T* + 1 cell types, as


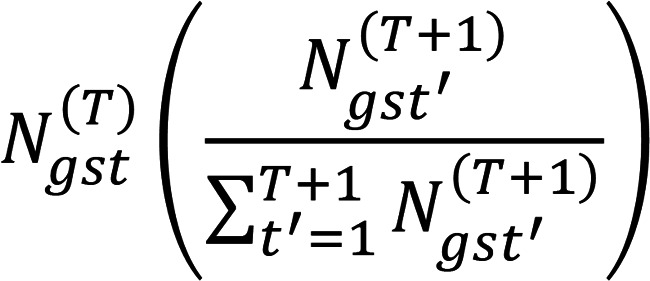
 (Equation 1)

We then estimated the number of total reads of cell type *t* among *T* cell types that were also assigned to cell type *t*′ among *T* + 1 cell types as the sum of this expression over every gene-sample combination, or


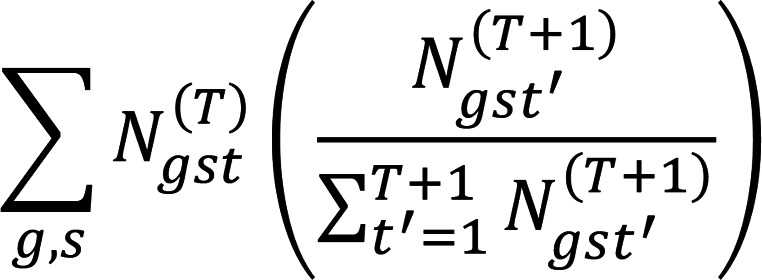
 (Equation 2)

This quantity represents transcripts that are preferentially assigned to cell type *t* over other cell types and that also constitute a substantial fraction of cell type *t*. For each cell type, we computed the proportion of each sample that comprised the cell type and computed cell types preferentially found in GBM over normal samples using a 2-tailed *t* test, using the Holm-Bonferroni method to correct for multiple comparisons.

Cell types were identified by comparing the transcriptome of each cell type with single-cell RNA-Seq data from a set of healthy control patients included in a publicly available dataset of peripheral blood single-cell data (22). Samples from patients in this dataset with COVID-19, which was the subject of the study for which these data was obtained, were excluded. The distance between the distribution of gene expression associated with each bulk cell type and the distribution associated with the annotated single-cell types was computed using the Hellinger distance


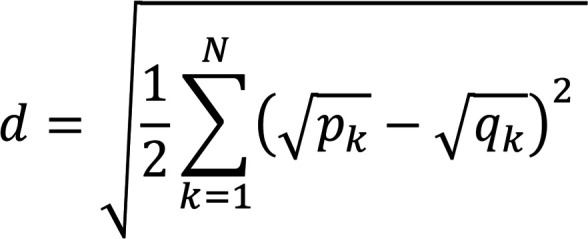
 (Equation 3)

where *N* is the total number of genes, numbered *k* = 1, …, *N*, and *p_k_* and *q_k_* are the expression of the gene in the bulk data and single-cell data, respectively. The single cell type closest to a given bulk cell type was selected as the correct annotation. In practice, several single cell types were nearly equally close in distance to each nonplatelet bulk cell type but were of the same hematopoietic lineage; therefore, in these cases, the cell type was annotated using the hematopoietic lineage (see Results). Hierarchical clustering was performed on the bulk cell types using the Hellinger distance to compare cell types, as described above. The same single-cell data set was used to approximate the number of RNA molecules per cell for a given type by taking the mean number of reads among all cells of each type. For cell type G (unknown type with high ribosomal protein counts), the mean read count of all cells in the data set excluding erythrocytes and platelets was used.

We also examined transcripts that were differentially upregulated in the platelet transcriptomes of patients with GBM compared with healthy controls. Analysis was performed using DESeq2 (20), and transcripts with a greater than 1.25 log_2_-fold increase in expression, at a significance level of *P* < 0.05, were selected. These genes were then clustered together using hierarchical clustering of the distribution of the transcripts of each gene in the deconvolution algorithm, using 2 to 60 cell types; using the notation above, the distance between 2 genes *g* and *g*′ is given by


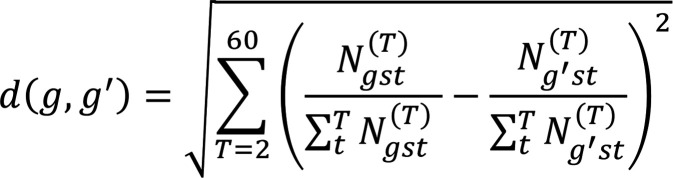
 (Equation 4)

That is, genes that tended to be allocated to the same cell type across deconvolution attempts for different numbers of cell types were more likely to be clustered together. For each gene, we analyzed the cell types to which the transcripts were assigned when performing deconvolution into 30 cell types. We also analyzed the share of each platelet cell type transcriptome that was constituted by the transcripts of each of these genes, as well as when performing deconvolution into 30 cell types.

The above algorithms were also used to analyze an RNA-Seq dataset of patients with NSCLC using GEO accession number GSE89843 (17). Processing was performed as described above, and deconvolution and analysis were performed using a dataset of healthy controls from the aforementioned studies combined with NSCLC samples.

To run the LDA algorithm in a supervised fashion, we used the implementation of the supervised LDA algorithm in tomotopy 0.12 (51). We first used the standard unsupervised LDA algorithm with *k* = 2, with α = 20 and β = 0.5, to deconvolute the data into 2 cell types corresponding to platelets and nonplatelets, running the algorithm for 2,000 steps. We then used the platelet cell type from the resulting deconvolution as input for the supervised LDA algorithm, using *k* = 30 cell types, α = 0.1, and β = 0.01, with a binary classifier set with μ = 0 and ν^2^ = 1, and setting the response variables to 0 for control samples and 1 for GBM samples. This was run for a total of 1,000 steps, and the inferred response value for each data sample in the training and validation sets was used for class prediction.

To compare patient follow-up samples and samples from patients with multiple sclerosis (both included in GEO accession number GSE156902) with our data set of samples of healthy controls and initial samples from patients with GBM, we used a “fold-in” technique to directly compare these additional samples with the ones analyzed by the initial pipeline. We used the same processing pipeline as above to generate a dataset of aligned reads. We then downsampled these samples by a factor of 10 to match the dilution factor used by CDSeq for the original samples and to make the analysis computationally tractable. We then ran 1,000 cycles of our own LDA algorithm, including both the new samples and the already analyzed samples, but only changing the cell type assignments of the new sample reads while keeping the cell type assignments of the previously analyzed samples. Thus, the new samples are “folded into” the previous analysis so that cell type annotations are identical and the new samples can be analyzed within the same framework as the previous samples. We used the same analytical pipeline to compare samples from patients with NSCLC brain metastases with patients with primary NSCLC.

### Mouse experiments.

GS 8-11 glioblastoma sphere cells, developed in our laboratory as described previously (52), 1 × 10^5^ per mouse, were implanted into the brains of 6- to 8-week-old Foxn1^nu^ mice (Charles River Laboratories), 1 week after guide screw placement, using a stereotactic apparatus under anesthesia. A minimum of 5 mice were included in each group. Animals in the treatment group that showed signs of distress or were moribund were euthanized, and blood was collected using an 18-gauge needle by cardiac puncture. Five weeks after tumor implantation, approximately 1 mL of blood was collected from each mouse in a BD Vacutainer yellow-top tube containing acid citrate dextrose to prevent platelet activation. Platelet-rich plasma (PRP) was collected by centrifuging the tubes at 100*g* for 15 minutes. The centrifuge was set to accelerations of 5 and 2 to avoid platelet activation. PRP was separated carefully from the other components of the blood and centrifuged at 1,000*g* for 10 minutes after the addition of 2 μL of 1 mM prostaglandin E_2_ to 2 mL of PRP. RNA was isolated from the resulting platelet pellets using the mirVana miRNA Isolation Kit (Invitrogen, catalog AM1560), followed by standard RNA-Seq library preparation and paired-end sequencing using NextSeq 2000 (Illumina). FASTQ files were processed as described above in *Deconvolution with CDSeq*.

The DSVD lung adenocarcinoma mouse model was intranasally induced with Ad-CMV-Cre (5 × 10^7^ plaque-forming units) as previously described (53). C57BL/6 mice were used for the study. Approximately 1 mL of blood was collected from each mouse by cardiac puncture using an 18G needle, 3 weeks after tumor implantation. The samples were stained with a panel of 13 barcoded CITE-Seq–compatible antibodies (Table 1). Briefly, a maximum of 1 million cells per sample were resuspended in 100 μL of cell staining buffer (2% BSA/0.01% Tween, PBS) with 10 μL of Fc receptor block (TrueStain FcX, BioLegend) for 10 minutes. This was followed by a 30-minute staining with the antibodies at 4°C. A concentration of 1 μg/100 μL was used for all the antibody markers used in this study. The cells were then washed 3 times with 1 mL of staining buffer, followed by centrifugation (350*g* for 5 minutes at 4°C) (54). Stained cells were then used for single-cell reverse transcription using 10x Genomics, and libraries were prepared as previously described (32). Briefly, cDNA amplification was performed in the presence of an antibody oligo-specific primer to increase the yield of antibody-derived tags (ADTs). The amplified cDNA was separated by solid-phase reversible immobilization size selection into cDNA fractions containing mRNA-derived cDNA (>300 bp) and ADT-derived cDNAs (<180 bp). Sequencing libraries were generated from the mRNA and ADT cDNA fractions, which were quantified, pooled, and sequenced on an Illumina NovaSeq platform.

CITE-Seq data were processed using Seurat v.4 pipeline (55). Platelets were selected from the single-cell dataset by visual inspection of the UMAP plot of the total data set, selecting the cell cluster that contained high levels of platelet markers, including *Ppbp* and *Pf4*, then removing cells with >2% ribosomal protein transcripts, >2% hemoglobin transcripts, >10% mitochondrial mRNA transcripts, or increased levels of *Malat1*. Platelet datasets from normal mice and mice with implanted tumors were integrated using the Seurat pipeline. The integrated data were then processed using the standard Seurat pipeline, with markers of Seurat clusters found using the FindConservedMarkers function.

### Statistics.

For analysis of rates of human transcripts in control and tumor-implanted mice, the mean was calculated and 95% confidence intervals were calculated using a bootstrap. Gene set enrichment analysis was performed using topGO (28), and *P* values were calculated using Fisher’s exact test. Differential expression testing *P* values were calculated using a Wald test, using the standard DESeq2 methods. Differences in expression levels and read counts in CITE-Seq data analysis were analyzed using a Wilcoxon test.

### Study approval.

All animal procedures were reviewed and approved by the Institutional Animal Care and Use Committee at NYU Grossman School of Medicine.

### Data availability.

The data used for the platelet deconvolution study are all publicly available datasets and include RNA-Seq data from GEO accession numbers GSE156902 and GSE89843. CITE-Seq data and RNA-Seq data with mice implanted with glioma stem cell lines are available at the GEO under the series GSE271072 and GSE271073. All supporting data are provided in the Supporting Data Values file.

## Author contributions

JMK designed the study and performed the analysis. JMK, ASM, KPLB, and EPS interpreted the data. JMK, RE, KPLB, and EPS wrote the manuscript. RE, MG, AS, ZYZ, YD, MS, TC, and SL performed experiments. KKW and BR provided guidance and study materials. RE did administration and management. All authors have reviewed and revised the manuscript.

## Supplementary Material

Supplemental data

Supporting data values

## Figures and Tables

**Table 1:**
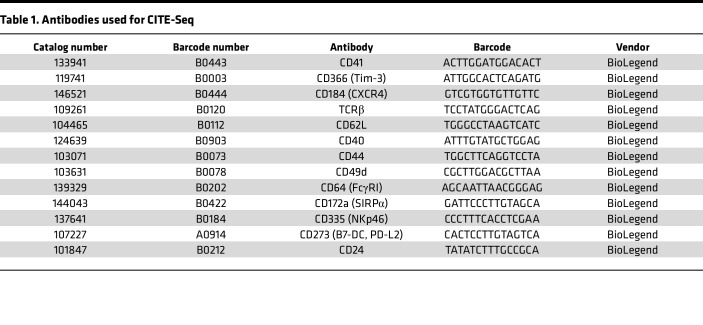
Antibodies used for CITE-Seq

## Version 1 08/27/2024

In-Press Preview

## Version 2 10/08/2024

Electronic publication
