# Supplementary material for: Deconvolution of the tumor-educated platelet transcriptome reveals activated platelet and inflammatory cell transcript signatures
Source: JCI Insight. 2024 Aug 27;9(19):e178719. doi: 10.1172/jci.insight.178719 (PMC11466191; doi:10.1172/jci.insight.178719)

**Supplemental Figure 1.** Deconvolution of the platelet transcriptome using CDSeq, with the number of cell types varying from 2 to 60. Each column represents the same set of reads that were deconvoluted into a different number of cell types. Flow from one column to the next represents an estimate of the repartitioning of reads into a larger number of cell types.

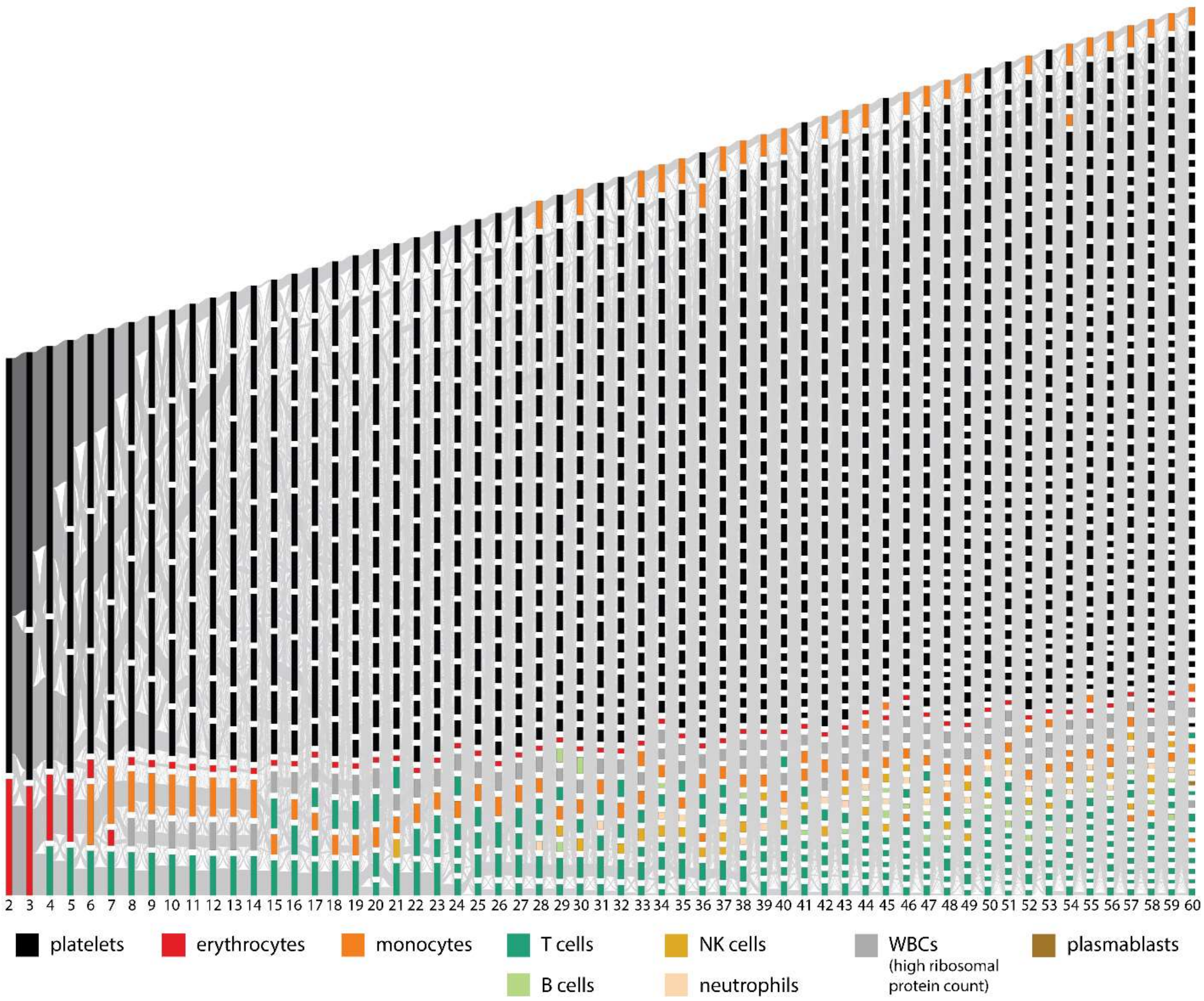

**Supplemental Figure 2.** Log-posterior of the CDSeq deconvolution model for 2 to 60 cell types.

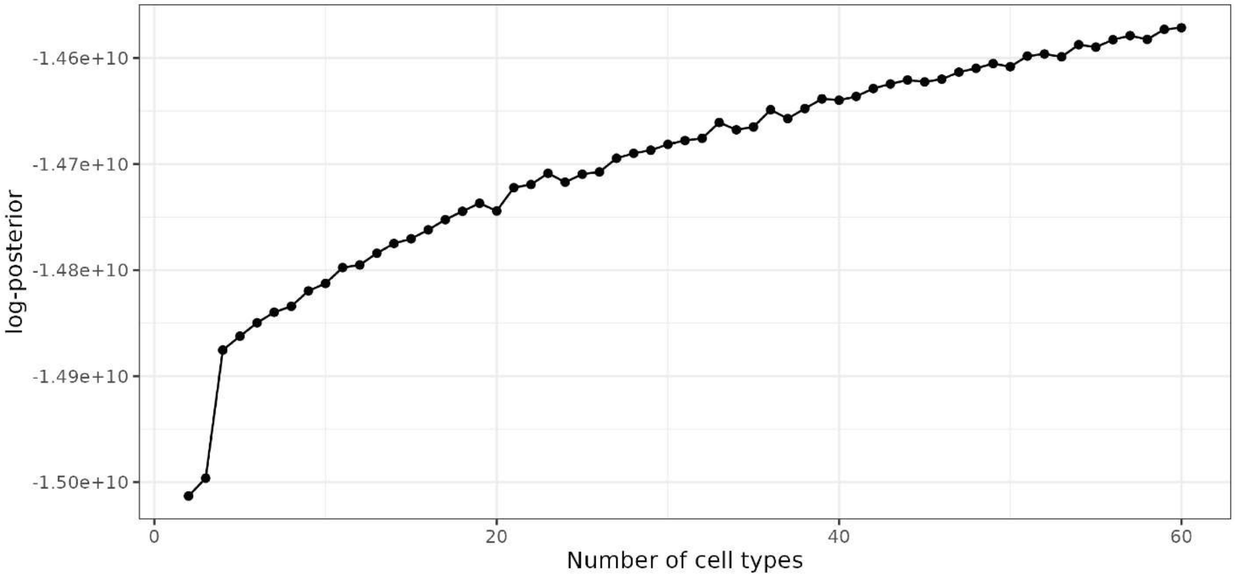

**Supplemental Figure 3.** The expression of ribosomal protein genes in single-cell data used by Jin et al. The circled cluster contains platelets.

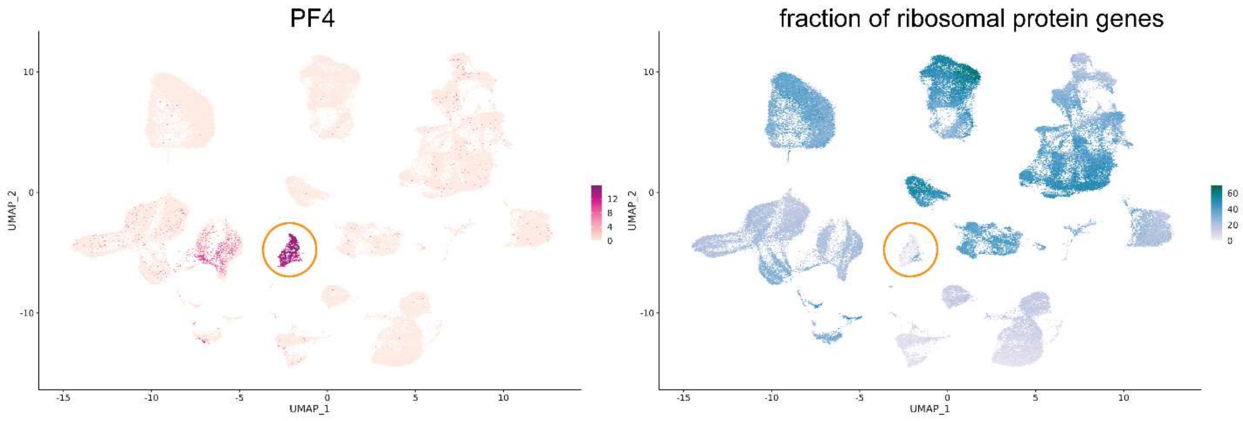

**Supplemental Figure 4.** Proportion of transcripts corresponding to non-platelet cell types in samples collected from different institutions.

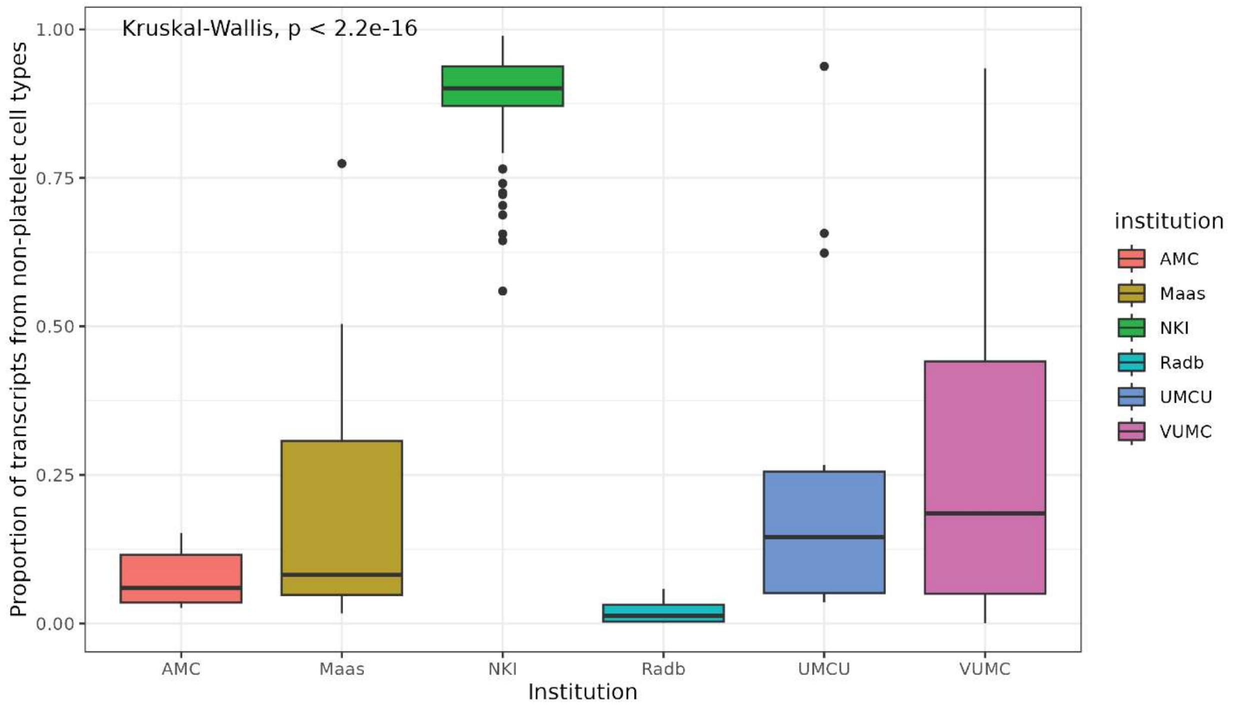

**Supplemental Figure 5.** Expression of platelet activation-related genes (*MYL9*, *MYH9*, *ITGA2B*, *GP1BB*, and *FLNA*), *RGS10* and *RGS18* in platelet cell types.

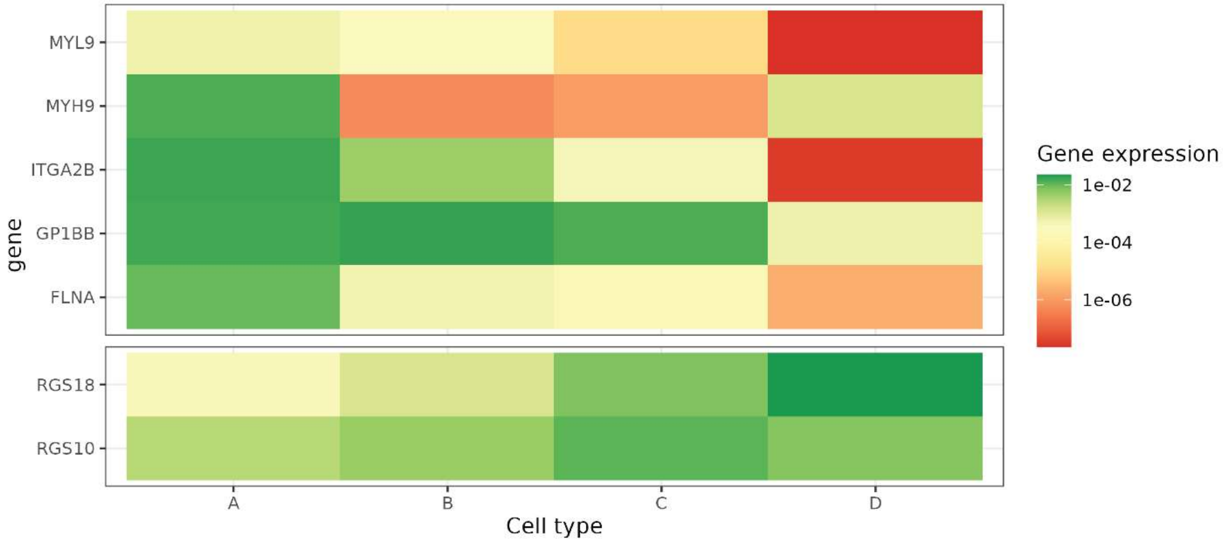

**Supplementary Figure 6.** Division of deconvolution analysis into samples with high circRNA fraction and low circRNA fraction. Compare to Fig. 3A.

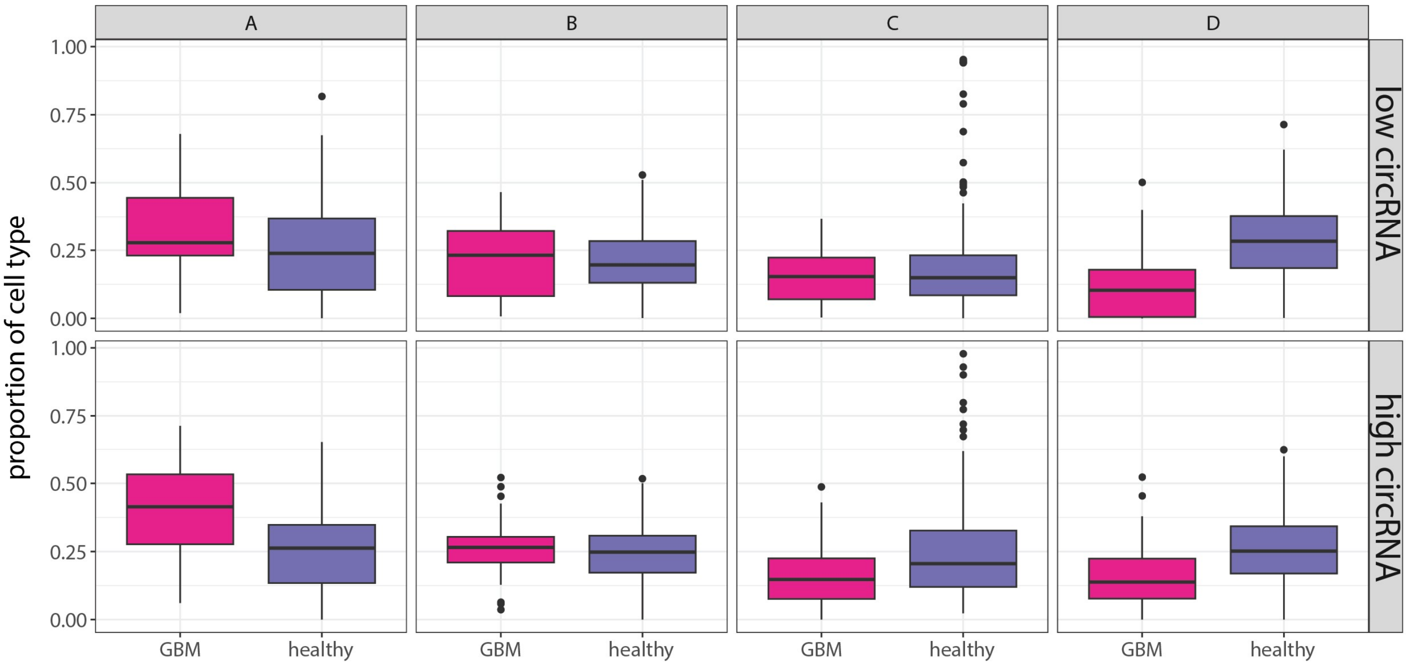

**Supplemental Figure 7.** Results of fold-in analysis for deconvolution of platelets from patients with multiple sclerosis (MS), based on deconvolution of samples from healthy controls and patients with GBM. (A) Results of deconvolution into 8 cell types, with 4 platelet subtypes, showing fraction of sample attributed to each subtype after non-platelets are removed. (B) Fraction of sample attributed to cell type 16, which is the cell type highly enriched in patients with GBM, as shown in Figure 5.

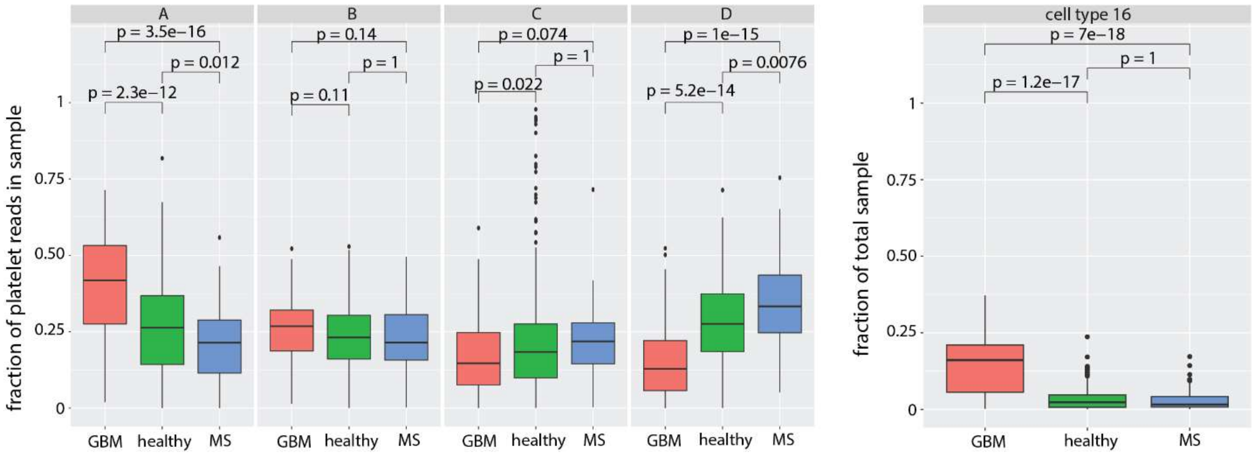

**Supplemental Figure 8.** Results of sample classification using supervised LDA algorithm for training and validation sets.

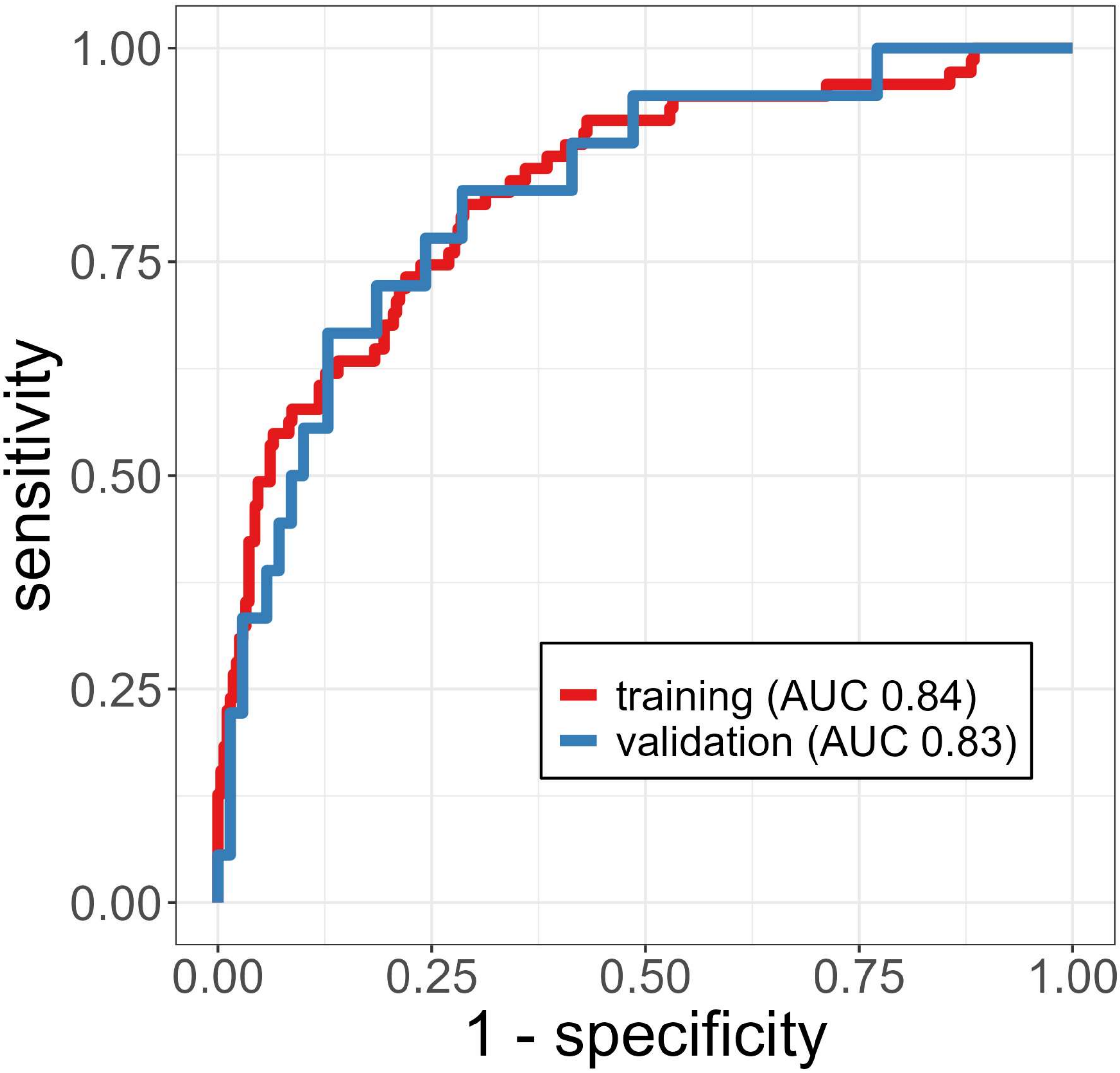

**Supplemental Figure 9.** Deconvolution of the platelet transcriptome using CDSeq for platelet samples derived from healthy controls and patients with non-small cell lung cancer. Compare to Fig. 2.

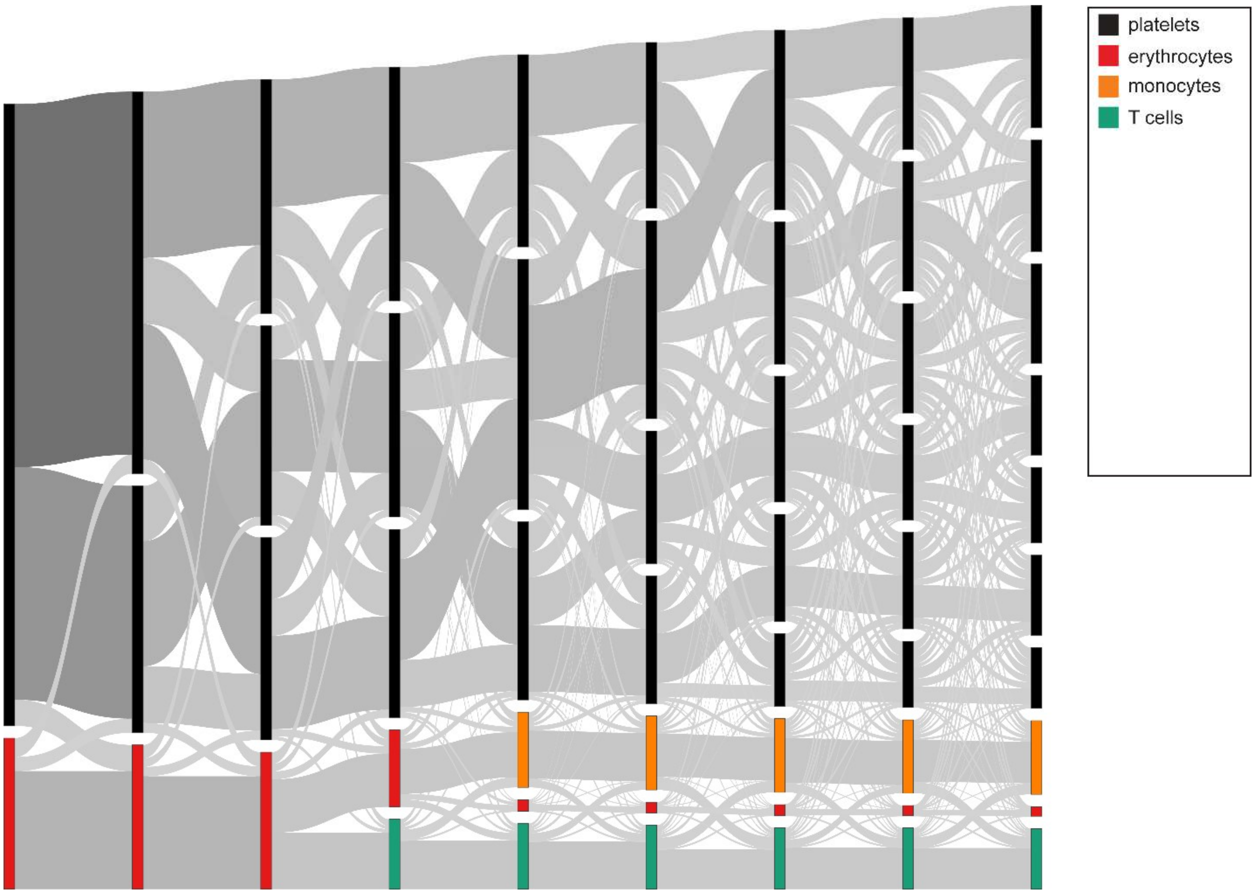

**Supplemental Figure 10.** Boxplot showing the fraction of each of the four platelet cell types in samples from healthy controls and patients with NSCLC, when samples were deconvoluted into 7 cell types. Compare to Fig. 3a for GBM.

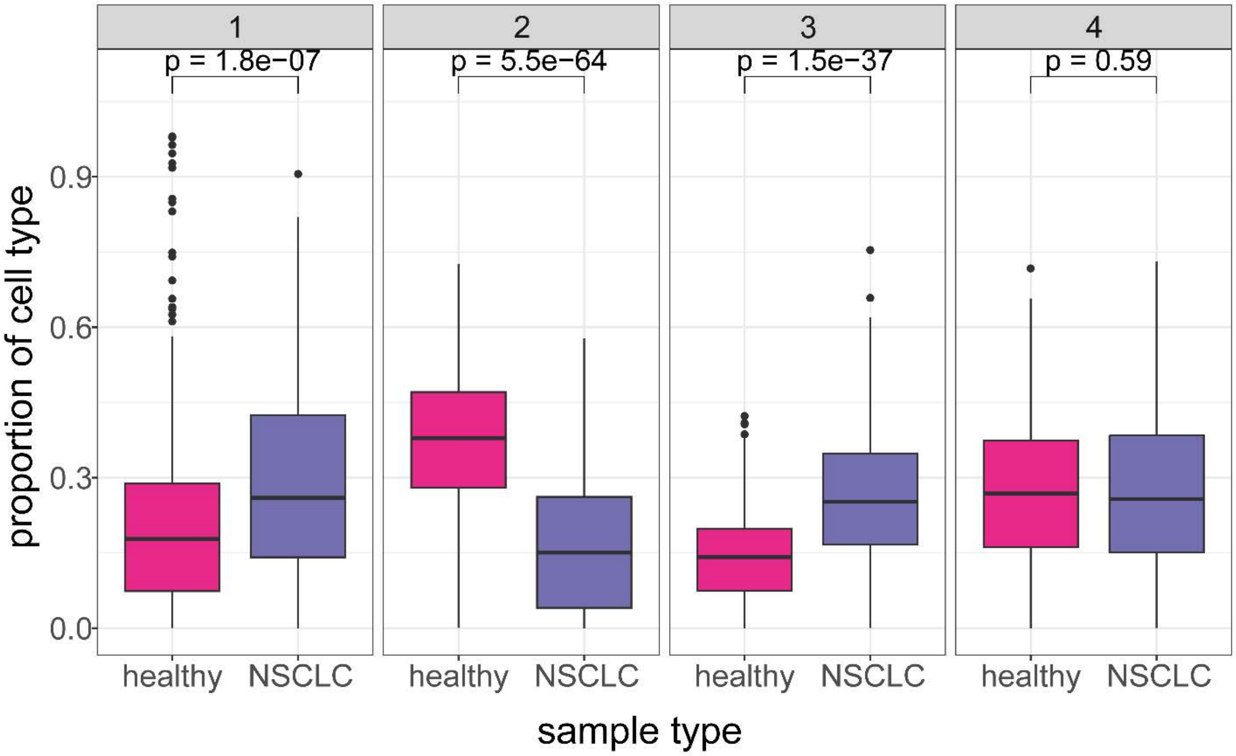

**Supplemental Figure 11**. (A) Clustering dendrogram, (B) distribution of genes among cell types when deconvoluting into 30 cell types, and (C) expression of genes among cell types, for genes upregulated in the platelet transcriptome of patients with non-small cell lung cancer. Platelet types are ordered by activation type, calculated based on the Hellinger distance from the most active platelet cell type after deconvolution into 7 cell types (the same platelet type which is present in higher proportions in samples from patients with NSCLC (per Supplemental Fig. 7). See Fig. 5 for a comparison in GBM. The asterisk corresponds to the subtype shown in Supplemental Fig. 10.

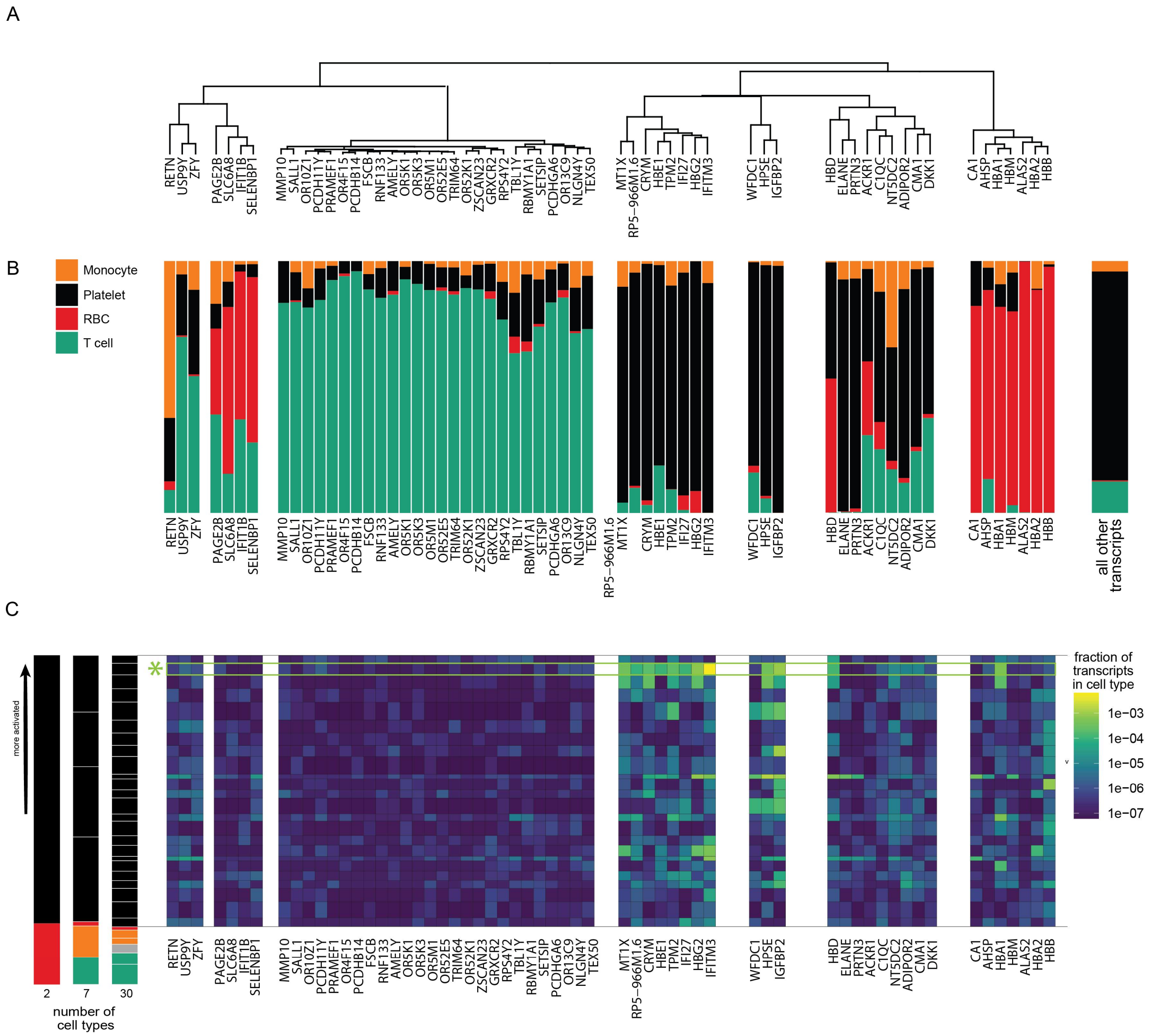

**Supplemental Figure 12.** Results of fold-in analysis for deconvolution of samples from patients with NSCLC brain metastases based on deconvolution of samples from healthy controls and patients with primary NSCLC tumors. (A) Results of deconvolution into 7 cell types, with 4 platelet subtypes, showing fraction of sample attributed to each subtype after non-platelets are removed. (B) Fraction of sample attributed to cell type 28, which is the cell type indicated by an asterisk in Supplemental Figure 11C.

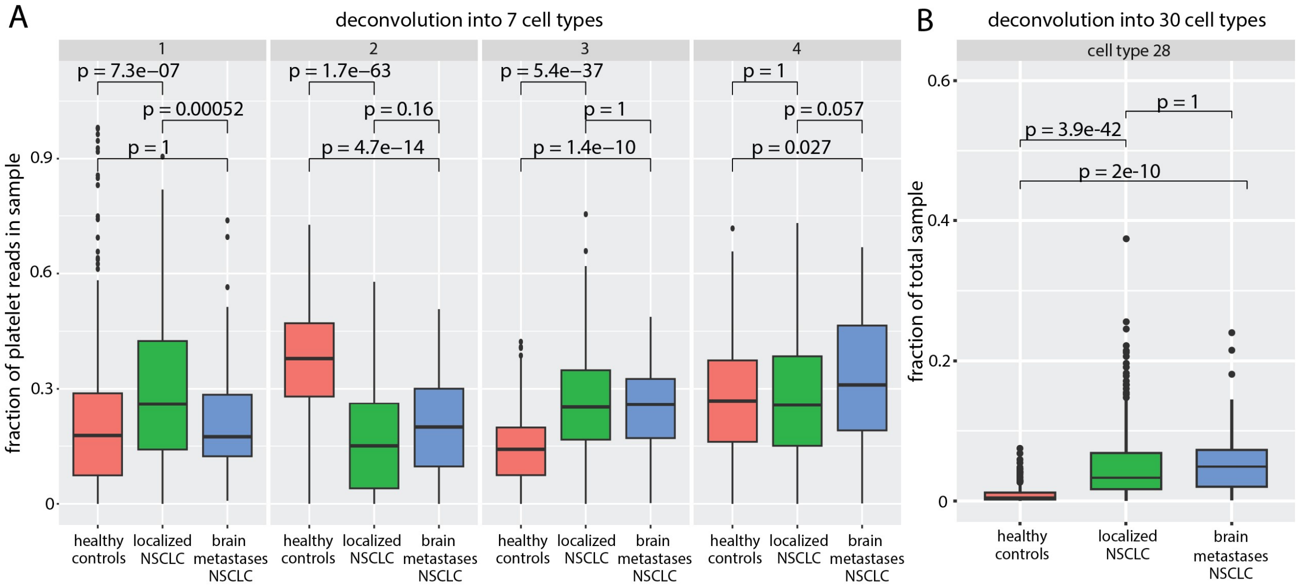

**Supplemental Figure 13.** (Top) Effect of variation of alpha and beta parameters on gene expression profiles. Fill color represents fraction of reads in the cell type with a given gene identity. Only genes with fraction of reads greater than $5 \times 10^{-4}$ in at least one cell type are included. (Bottom) Effect of variation of alpha and beta parameters on proportions of all cell types in each sample.

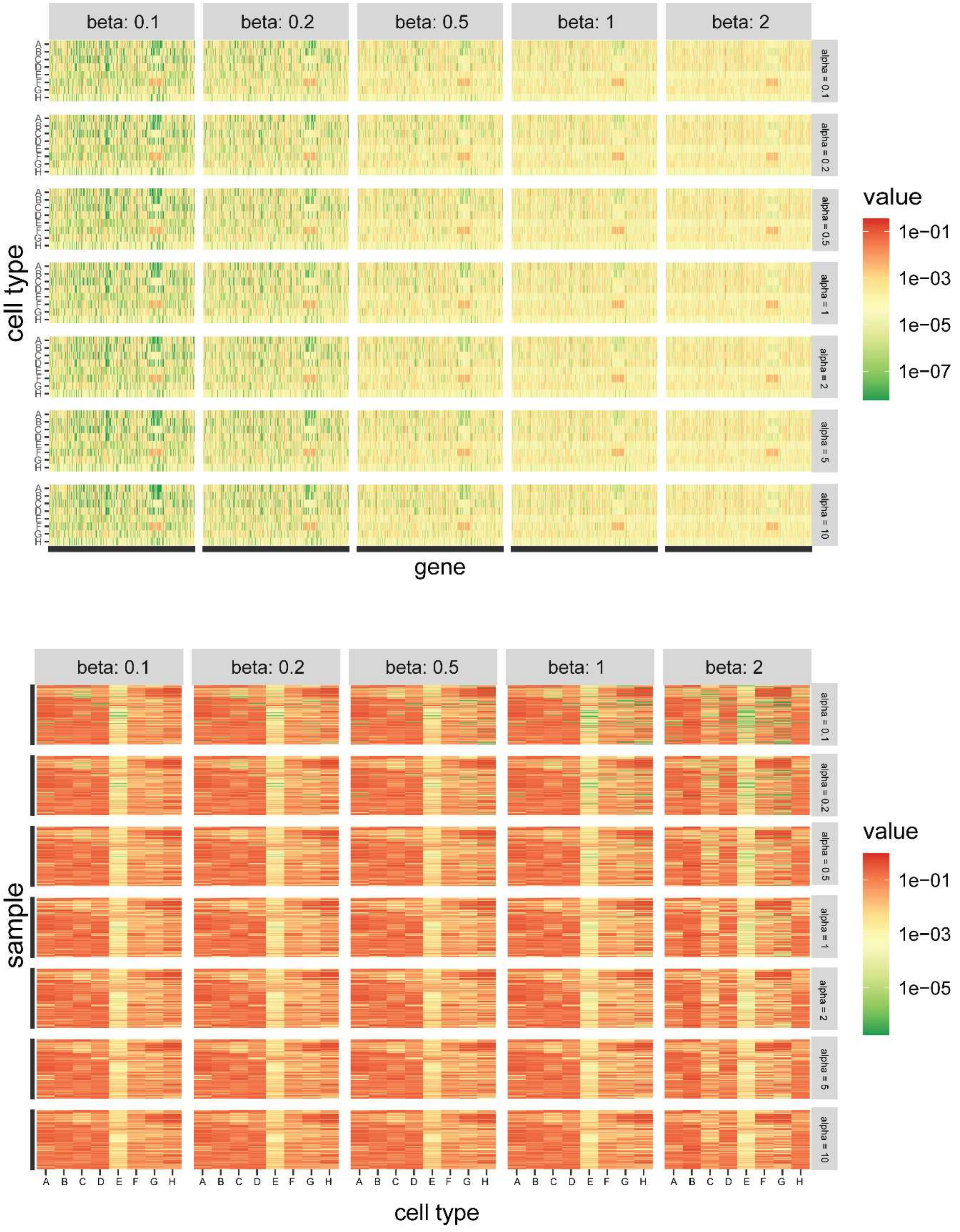

Supplement: Supplemental data [file jciinsight-9-178719-s063.pdf]
